# Quality Control Platform for the Standardization of a Regenerative Medicine Product

**DOI:** 10.3390/bioengineering9040142

**Published:** 2022-03-28

**Authors:** Silvia Zia, Barbara Roda, Chiara Zannini, Francesco Alviano, Laura Bonsi, Marco Govoni, Leonardo Vivarelli, Nicola Fazio, Dante Dallari, Pierluigi Reschiglian, Andrea Zattoni

**Affiliations:** 1 Stem Sel srl, 40127 Bologna, Italy; barbara.roda@unibo.it (B.R.); pierluigi.reschiglian@unibo.it (P.R.); andrea.zattoni@unibo.it (A.Z.); 2 Department of Chemistry “G. Ciamician”, University of Bologna, 40126 Bologna, Italy; 3 Unit of Histology, Embryology and Applied Biology, Department of Experimental, Diagnostic and Specialty Medicine, University of Bologna, 40126 Bologna, Italy; chiara.zannini5@unibo.it (C.Z.); francesco.alviano@unibo.it (F.A.); laura.bonsi@unibo.it (L.B.); 4 Reconstructive Orthopaedic Surgery and Innovative Techniques—Musculoskeletal Tissue Bank, IRCCS Istituto Ortopedico Rizzoli, 40136 Bologna, Italy; marco.govoni@ior.it (M.G.); leonardo.vivarelli@ior.it (L.V.); dante.dallari@ior.it (D.D.); 5 Technology Transfer Office, IRCCS Istituto Ortopedico Rizzoli, 40136 Bologna, Italy; nicola.fazio@ior.it

**Keywords:** adipose stem cells, SVF, label-free separation, quality control protocol

## Abstract

Adipose tissue is an attractive source of stem cells due to its wide availability. They contribute to the stromal vascular fraction (SVF), which is composed of pre-adipocytes, tissue-progenitors, and pericytes, among others. Because its direct use in medical applications is increasing worldwide, new quality control systems are required. We investigated the ability of the Non-Equilibrium Earth Gravity Assisted Dynamic Fractionation (NEEGA-DF) method to analyze and separate cells based solely on their physical characteristics, resulting in a fingerprint of the biological sample. Adipose tissue was enzymatically digested, and the SVF was analyzed by NEEGA-DF. Based on the fractogram (the UV signal of eluting cells versus time of analysis) the collection time was set to sort alive cells. The collected cells (F-SVF) were analyzed for their phenotype, immunomodulation ability, and differentiation potential. The SVF profile showed reproducibility, and the alive cells were collected. The F-SVF showed intact adhesion phenotype, proliferation, and differentiation potential. The methodology allowed enrichment of the mesenchymal component with a higher expression of mesenchymal markers and depletion of debris, RBCs, and an extracellular matrix still present in the digestive product. Moreover, cells eluting in the last minutes showed higher circularity and lower area, proving the principles of enrichment of a more homogenous cell population with better characteristics. We proved the NEEGA-DF method is a “gentle” cell sorter that purifies primary cells obtained by enzymatic digestion and does not alter any stem cell function.

## 1. Introduction

Somatic cell therapy is a growing application in advanced therapy medicinal products (ATMPs). Cellular products are currently used to treat infections, autoimmunity, cancers, metabolic diseases, and tissue degeneration, as well as in tissue-engineering approaches [1,2,3].

Regulatory compliance requires new technologies to standardize and check the quality of the production process of cell-based therapy products. Definition of the quality of cell-based therapies is challenging given the complexity and novelty of the products. Cell-based therapy products must be developed in designated clean environments equipped with expensive devices that require high maintenance. In addition, manual cell culture practices are not able to provide the high throughput needed for the development of new cellular products. Standardized methods for sample handling and storage will also maximize the success and reproducibility of cell-based therapy products.

Standardized protocol design is important for the development of cell-based therapy products and to improve basic research, as well. The tools and technologies that are being developed for cell-based therapy could be acquired for research laboratories to improve the standardization of techniques and data reproducibility [4,5].

Among cellular products, stem cells (SCs) are one of the major candidates for regenerative medicine due to their therapeutic properties based on stimulating host tissue regeneration through paracrine secretion of a series of biomolecules that both stimulate the proliferation of tissue-specific progenitors and induce localized immunomodulation [6,7].

SCs are distributed in all tissues, even though in vivo localization is not well defined because of the absence of a specific protein to distinguish them from progenitors or more differentiated cells. Moreover, the lack of homogeneity in pluri- and multi-potent SCs severely hinders definition and standardization for successful stem cell-based therapies. In the last decade, adipose tissue (AT) has been proven to be an increasingly attractive source of mesenchymal stem cells (MSCs) for tissue regeneration because of ease of accessibility in large quantities and use of non-invasive techniques. Isolation of adipose-derived MSCs (ASCs) by classical enzymatic digestion gives rise to a heterogeneous cell population containing all cells from the stromal vascular fraction (SVF). In order to use them for clinical applications, homogeneous preparations are strongly recommended.

To obtain a sufficient number of homogeneous SCs, effective sorting methods are needed. Cell-type-specific markers, such as cell surface proteins, used in fluorescence-activated cell sorting (FACS) and magnetic-activated cell sorting (MACS) technologies are limited and often recognize multiple members of a SC lineage. SC recovery and functionality are also affected by labelling [8,9]. Preserving cell integrity and functional characteristics and not inducing stress responses are current mandatory requirements in the sorting process. Methodologies using a fluorescence-activated cell sorter can lead to sorter-induced cell stress (SICS) [10], where pressure, mechanical forces, temperature, and laser irradiation can alter cells. Therefore, new approaches for cell sorting that are “gentler” have been investigated. Methods that exploit differences in biophysical cell characteristics are, therefore, promising when it comes to identifying and sorting homogeneous SC subpopulations [11]. Microfluidic techniques to dissect preparation composition have been already used for decades in pharmaceutical companies [12]. Among others, label-free techniques are ideal for their dual roles: analysis of the sample and sorting ability. The use of a microfluidic system forces cells to flow through plastic devices using biocompatible liquid. Even though material and flow rate are designed to not alter cell functionality, more studies are needed to prove their non-reactiveness.

The purpose of this work is to analyze freshly isolated SVF cells using a new label-free technology which exploits the Non-Equilibrium Earth Gravity Assisted Dynamic Flow Fractionation (NEEGA-DF) principles. Injected cells were separated based solely on their physical characteristics, such as dimension, morphology, density, and membrane rigidity. Cells with different morphological features can reach a specific position across the channel width, and, due to the parabolic flow profile, morphologically different cells acquire well-defined velocities and, therefore, can be separated. We previously demonstrated the ability to selectively fractionate MSCs from different sources [13] and from raw samples [14]. The separation process avoided cell contact, adhesion to the separation device, and cell–cell aggregation by using in-flow cell injection. Moreover, the flow rate values typically applied guaranteed low shear stress on cells. We established a protocol that analyzed freshly derived SVF cells. In particular, we focused on validation of the label-free system as an inert sorting process, as we analyzed the antigen surface protein phenotype, differentiation towards mesenchymal lineages, and immunomodulation ability of ASCs derived by the NEEGA-DF method.

## 2. Materials and Methods

### 2.1. Adipose Tissue Collection and Processing

A small amount of adipose tissue was collected as waste material from 4 cadaver donors during a routine procedure of musculoskeletal tissue processing performed by the accredited public non-profit Musculoskeletal Tissue Bank of the IRCCS (Istituto Ortopedico Rizzoli, Bologna, Italy; EU TE Code: IT000096). These samples were used for validation procedure purposes, according to the National Transplant Center guidelines. The isolation of the adipose-derived stem cells (ASCs) was performed as follows: tissue was mechanically minced and digested using a solution of 0.075% collagenase type II (Sigma-Aldrich, St. Louis, MO, USA) in PBS (Lonza Group, Rome, Italy) and was then incubated for 30 min at 37 °C in agitation. The sample was then centrifuged at 1500 rpm for 5 min, and the stromal cell pellet was recovered, resuspended, and filtered using a Whirl-Pak^®^ Filter Bag for Homogenizer Blenders (VWR International, Milan, Italy) to remove extracellular matrix components. Recovered cells were counted and then divided into three parts: a fraction was immediately analyzed by flow cytometry for phenotype; a second fraction was expanded in vitro at a plating density of 20.000 cells/cm^2^ in DMEM high glucose supplemented with 10% fetal bovine serum (FBS), 2 mM L-glutamine, and 1% penicillin streptomycin solution (all solutions from Gibco, Rodano, Italy); the third fraction was analyzed using the NEEGA-DF technique.

### 2.2. Characterization Adipose Tissue

A small piece of adipose tissue was fixed in 10% formalin for 30 min, and then it was washed two times in PBS for 10 min to remove the fixative solution. The tissue pieces were then placed on a coverslip and incubated with FITC-phalloidin primary antibody (1:100) in PBS for one hour and then washed two times with PBS to visualize the F-actin cytoskeleton. The tissue was placed on a coverslip and mounted using DAPI mounting medium. The tissue was visualized using a Nikon Inverted Fluorescent Microscope (Nikon Instruments, Amsterdam, The Netherlands), and images were captured with 10 and 20× magnification objectives.

SVF and F-SVF freshly isolated cells were plated at a density of 2000 cells/10 cm^2^ in expansion medium consisting of DMEM high glucose, 10% foetal bovine serum, 2 mM L-glutamine, and 1% penicillin and streptomycin (all reagents from GIBCO, Rodano, Italy) and cultured for 20 days to monitor colony-forming unit (CFU-F) ability. The cells were then fixed in 10% formalin for 10 min, washed in PBS, and colored with methyl violet to visualize colonies. The staining solution was removed, and the fixed colonies were washed two times with PBS. Images of colonies in SVF and F-SVF conditions were captured using a Leica Labovert FS Inverted Microscope (Leica Microsystems, Milano, Italy).

### 2.3. Separation Device and Method

#### 2.3.1. Label-Free Separation Device

**Figure 1:**
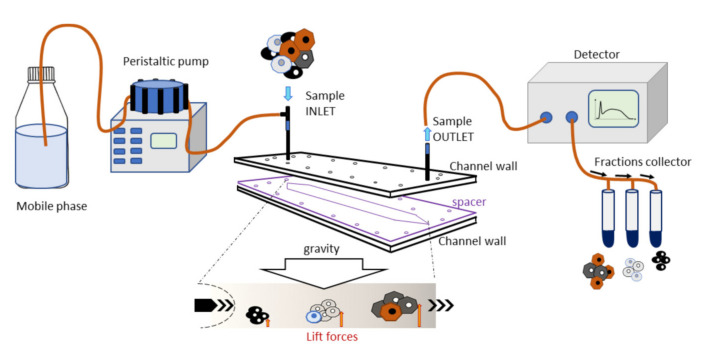
Graphical representation of the separation device. The separation device was connected to a peristaltic pump at the inlet and a UV detector at the outlet. Separated cells exited the detector and were collected in different tubes. Cell samples were inserted and cells, based on their physical characteristics, reached a specific position across the channel. Bigger and denser cells were the first to exit, followed by the smaller ones.

This instrument consists of a fluidic system and a biocompatible capillary separation device implementing a patented technology (IT1371772, US8263359, and CA2649234). The separation device is made of inert and biocompatible plastic material with a 40 cm length, a 4 cm width, and a 250 µm thickness connected to the fluidic system. At the fractionation device outlet, a UV/Vis detection system is connected to monitor the elution process, generating a recorded plot of the eluted cell as a function of time (fractogram). A fraction collector is connected to the separation device (Figure 1). The instrument was placed inside a laminar flow cabinet to provide sterile working conditions.

#### 2.3.2. Fractionation Principle and Procedure

The separation was obtained in a rectangle-shaped capillary device that was 4 cm wide and 250 µm high, where cell suspensions were eluted through a laminar flow of mobile phase. The injected cells reached a specific position across the channel width during transportation due to the combined action of gravity acting perpendicularly to the flow and opposing lift forces that depended on the morphological features of the sample. The cells at a specific position in the channel acquired well-defined velocities and were, therefore, eluted at specific times. Cell suspension was injected into the system at a flow rate of 1 mL/min.

The fractionation procedure involved, at first, the decontamination of the fractionation system by flushing with 3% chloride hypochlorite at a 1 mL/min flow rate. Next, the system was washed copiously with sterile, demineralized water at the same flow rate. Cells, MSCs in particular, can adhere to plastic: to block non-specific interaction sites on the plastic walls, the fractionation system was flushed at 0.5 mL/min with a sterile coating solution made of 1% bovine serum albumin (BSA, Sigma-Aldrich, St. Louis, MO, USA) dissolved in PBS. Finally, it was filled with a sterile mobile phase consisting of 0.1% BSA dissolved in PBS. All solutions were provided by Stem Sel, Ltd. (Bologna, Italy).

For the study, cells from the SVF were diluted to a final concentration of 6x10^6^ cells per ml and 100 µL was injected. The cells were re-dispersed 3 times to homogenize the suspension and were eluted at a flow rate of 1 mL/min. Fractionated cells (F-SVFs) were collected and biologically characterized to define proliferation rate, viability, surface markers, and multi-differentiation abilities. Unfractionated cells were used as reference control (SVF).

#### 2.3.3. Morphological Analysis

To perform morphological analysis of the heterogeneous population, one run for each sample was divided into 3 fractions (F1: 4–6 min; F2: 6–11 min; F3: 11–14 min), and the cells were collected and placed in a petri dish to evaluate shape in a suspension state. Pictures were obtained, and post-processing analyses on geometrical features (diameter, aspect–ratio, and circularity) were performed using free ImageJ software for image analysis (free software, NIH).

### 2.4. Immunomodulation Test

The immunomodulatory potential of ASCs on activated peripheral blood mononuclear cells (PBMCs) was studied using a co-culture system of ASCs and PBMCs with a ratio of 10:1 in RPMI medium with 10% FBS (Lonza Group, Rome, Italy). The PBMCs were isolated from healthy donors by density gradient centrifugation with Ficoll-Paque (Sigma-Aldrich, St. Louis, MO, USA) and activated by the addition of phytohemagglutinin (PHA, 1 μg/mL, Sigma-Aldrich, St. Louis, MO, USA). After an incubation of 72 h, the PBMCs were fixed with 70% ethanol and stained with propidium iodide at a concentration of 5 µ/mL (Beckman Coulter, Cassina de’ Pecchi, Milano, Italy) at room temperature for 10 min for cell cycle analysis by flow cytometry. PBMCs without PHA stimulation and PBMCs activated by PHA were analyzed as negative and positive controls, respectively. The immunomodulatory activity of the ASCs was also analyzed by BrdU incorporation in the PBMCs using an ELISA Kit according to the manufacturer instructions (BrdU Cell Proliferation ELISA Colorimetric Kit, Roche, Basel, Switzerland). All experiments were conducted using ASCs derived by NEEGA-DF selection (F-SVFs) directly plated from the SVF (SVF).

### 2.5. Proliferation Test

ASCs isolated and sorted from passages 1 to 6 were analyzed for their cumulative population doubling. In every passage, cells were harvested and counted by Trypan blue exclusion dye using the following formula: LOG10(recovered cells)-LOG10(initial cells)/LOG(2).

### 2.6. Flow Cytometry Analysis

The immunophenotypical profile of the freshly isolated and sorted ASCs was analyzed by flow cytometry for hematopoietic markers CD34-PE and CD45-FITC; endothelial-perivascular markers CD31-PE, CD146-PE; and mesenchymal markers CD44-FITC, CD73-PE, CD90-PC5; as well as CD105-PE antibodies. 100,000 SVF and F-SVF cells were divided in a single tube and incubated with 1% BSA in a PBS solution for 30 min. The cells were then centrifuged at 300 g for 5 min and incubated for 30 min with a specific antibody. Finally, the cells were centrifuged and resuspended in 200 µL PBS for reading using a FACS Canto flow cytometer (BD Biosciences, Milan, Italy).

### 2.7. Differentiation Potential

The stemness potential of the isolated ASCs was tested by differentiation potential towards mesenchymal lineages. Expanded SVF and F-SVF cells from passages 3–4 were trypsinized and plated for a differentiation assay.

Adipogenic differentiation: 20,000 cells/cm^2^ were plated and, 24 h later, were cultured with differentiation medium (Human Mesenchymal Stem Cell (hMSC) Adipogenic Differentiation Medium, Lonza Group, Rome, Italy) following manufacturer instructions. After 14 days, the cells were fixed in 10% formalin for 15 min and stained for 15 min using Oil Red O to visualize fat droplets. Quantification of the dye was performed using 100% isopropanol for 15 min in agitation. The solution was then read at 495 nm.

Osteogenic differentiation: 7500 cells/cm^2^ were plated and, 24 h later, were cultured with differentiation medium (StemPro Osteogenesis Differentiation Kit, Gibco, Rodano, Italy). After 21 days, the cells were fixed in 10% formalin for 15 min and stained for 30 min using Alizarin Red S to visualize the extracellular matrix deposition layer over the cell layer.

Angiogenic assay: 200,000 and 400,000 cells/cm^2^ were seeded in tissue culture flasks as control and induced conditions, respectively. The cells were cultured in DMEM high glucose with 10% FBS for 24 h, and on the following day, the medium was replaced with a differentiation medium composed of DMEM high glucose, 2% FBS, and VEGF 50 ng/mL (Sigma-Aldrich, St. Louis, MO, USA). The medium was replaced every two days, and, after 6 days, the cells were trypsinized. A total of 15,000 cells were transferred in a 96-well plate precoated with ice-cold Matrigel (Basement Membrane Matrix, BD Biosciences, Milan, Italy) and cultured for 24 h in DMEM high glucose. Pictures were obtained using a camera every two hours to observe tube-like formation.

### 2.8. Statistical Analysis

All data were plotted and analyzed by GraphPad Prism software using two-way ANOVA multiple comparison (* *p* < 0.05, ** *p* < 0.01, *** *p* < 0.001, **** *p* < 0.0001).

## 3. Results

### 3.1. ASC Isolation and NEEGA-DF Analysis

**Figure 2:**
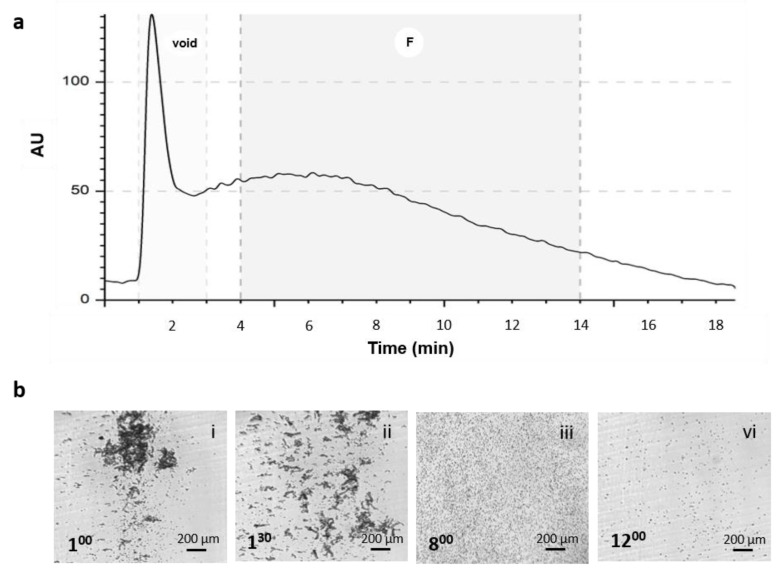
Analysis of fresh stromal vascular fraction (SVF) sample using the Non-Equilibrium Earth Gravity Assisted Field Flow Fractionation (NEEGA-DF) method. (**a**) Representative fractogram of fresh SVF cells showing absorbance versus time of analysis. (**b**) Representative images of eluting cells collected at different times of analysis. In particular, it shows cells eluting in the initial phase (void), when debris, dead cells, red blood cells, and extracellular matrix complexes exited the channel (**i**,**ii**), as well as single cells later in the analysis belonging to the fraction F (**iii**,**iv**).

Adipose tissue consists of mature adipocytes and cells from the stromal vascular fraction (SVF). In particular, the SVF contains various hematopoietic, vascular, and mesenchymal stromal cells (MSCs); the latter are usually identified as adipose-derived stromal cells (ASCs). SVF cells from cadaveric donors were isolated by enzymatic digestion with collagenase type II and then immediately analyzed by the NEEGA-DF method. The cells were analyzed in order to obtain a fingerprint of the biological sample using the data output: the live fractogram. The live fractogram represented the eluted cells vs. the elution time, and it showed the sample complexity (Figure 2a). Fractograms were similar among the samples, with a high initial peak (void) composed of debris, extracellular matrix components, dead cells, and red blood cells (Figure 2b(i),(ii)), followed by a wide signal band from the third minute until the end of the analysis around the fifteenth minute (Figure 2b(iii),(iv)). Cells from the void were excluded to enrich the vital and proliferative component, the fraction titled F, which eluted from the fourth until the fourteenth minute.

F-SVF cells were collected and then equally divided in two groups: the first half for phenotypic characterization by flow cytometry, and the second half for expansion in vitro to monitor plastic adhesion features, morphology, proliferation, immunomodulation ability, and differentiation potential towards mesenchymal lineages.

The F-SVFs after expansion were re-injected in the fractionation device; the typical profile for ASCs derived from adipose tissue was observed (Figure S1). Elution times for the fresh SVF and expanded ASCs were similar, confirming the enrichment in MSCs after NEEGA-DF fractionation of the freshly harvested SVF sample.

### 3.2. Morphological Characteristics of Sorted SVF Cells

**Figure 3:**
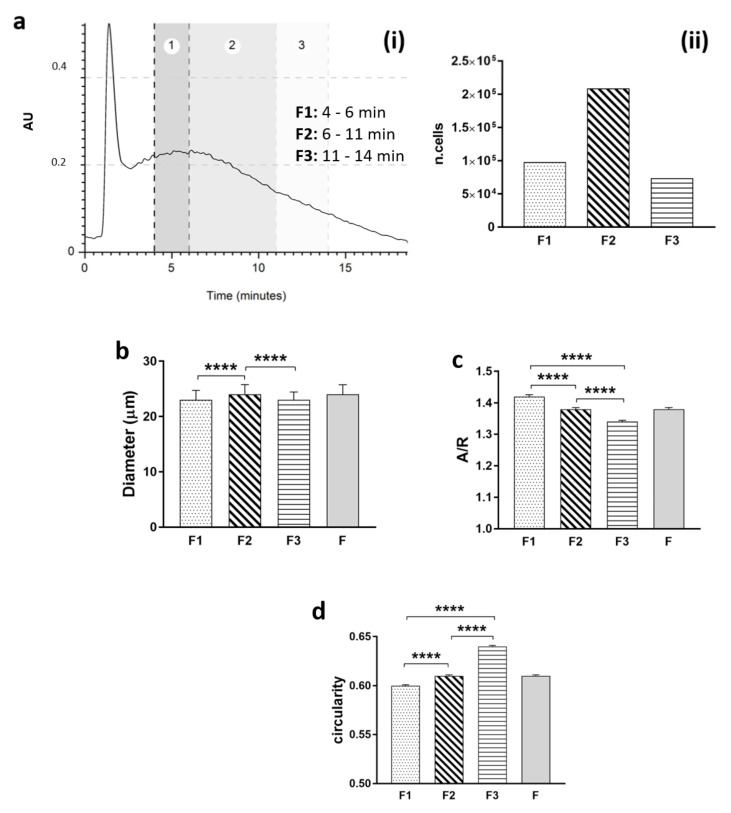
Based on stromal vascular fraction (SVF) fractograms, (**a**) cells were divided and collected at three time intervals (F1: 4–6 min; F2: 6–11 min; F3: 11–14 min). (**i**) Cells from each fraction were counted, (**ii**) bright field microscopic images were post-processed using ImageJ software, and the averages of diameter (**b**), aspect–ratio (**c**), and circularity (**d**) were plotted (*t*-test; **** *p* < 0.0001).

Post-processing analysis for data visualization allowed us to better interpret the cell composition of freshly isolated SVF cells. Collected cells in suspension were counted and geometrical analysis was performed. The averages of the physical parameters for selected fractions (F1, F2, and F3) were recapitulated by column statistics (Figure 3). The majority of cells eluted in F2 had a diameter of 24 µm, compared to 23 µm from F1 and F3 cells (Figure 3b). F1 cells showed a higher value of aspect–ratio, which describes the overall shape of the cells in terms of how elongated cells are (Figure 3c). The higher value could also be generated by cells in an aggregate state.

On the contrary, F3 cells showed a lower aspect–ratio of 1.34 and a higher circularity of 0.64, which describes how ruffled or irregular the membranes of cells are (Figure 3d). As seen by the circularity graph, it is evident how the cells acquired a more regular membrane with the increase in the time of elution, implying a separation of cells with more defined characteristics.

### 3.3. Phenotype of Sorted Cells

**Figure 4:**
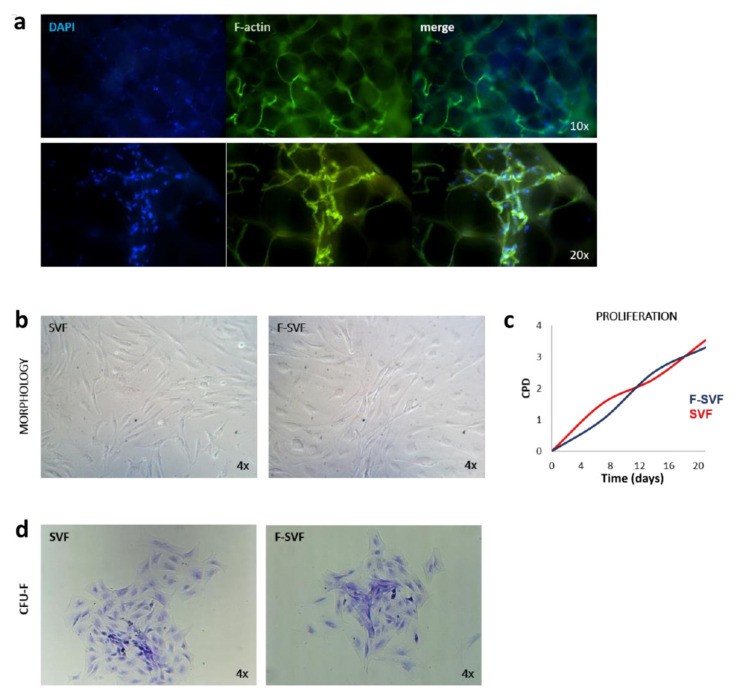
Adipose tissue skeleton structure was visualized by phalloidin staining: (**a**) representative images of cells derived by stromal vascular fraction (SVF) and the fraction derived vascular fraction (F-SVF). In both conditions, the cells had similar morphology to a fibroblastoid shape (**b**); the cells show the same proliferation ability by calculation of cell population doubling time (**c**); SVF and F-SVF cells demonstrated to have the same ability to form colony forming unity fibroblasts (CFU-F) (**d**).

Adipose tissue before enzymatic digestion showed actin fibers surrounding adipocytes and cells in the interstice (Figure 4a).

When the cells were collected from the device, the results were sterile and vital with the same adherent morphology compared to SVF cells (Figure 4b). Proliferation was monitored by cell population doubling time and showed no difference between the two groups (Figure 4c). One of the characteristics to define mesenchymal stem cells is their ability to form CFU-F and naïve cells (SVFs), and the analyzed cells (F-SVFs) showed the same ability to form CFU-F (Figure 4d).

**Figure 5:**
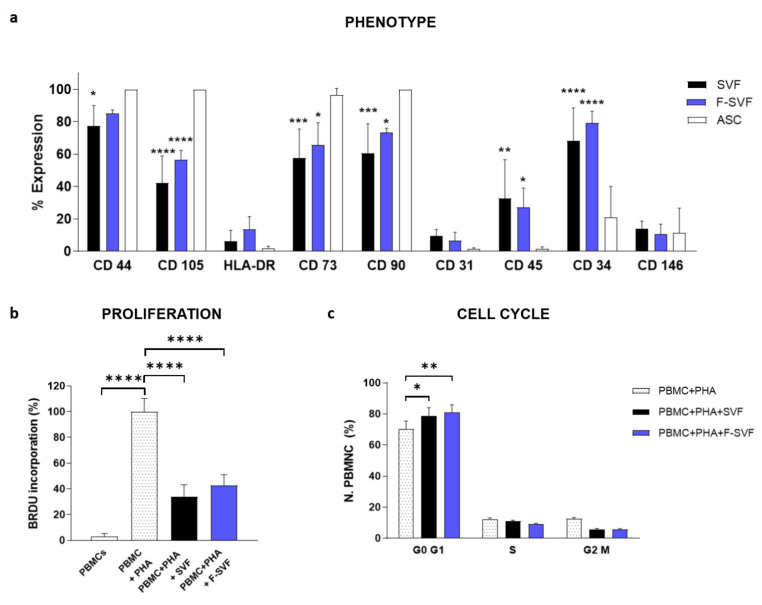
Phenotype characterization of stromal vascular fraction (SVF) and the fraction derived stromal vascular fraction (F-SVF) cells by flow cytometry. Mesenchymal markers were slightly more expressed in the F-SVF, proving an enrichment of the mesenchymal component of the SVF due to the label-free sorting (**a**). SVF and F-SVF cells showed the same immunomodulatory ability to inhibit the proliferation of PHA-stimulated PBMCs by BrdU assay (**b**). Moreover, it was shown that SVF and F-SVF cells arrested stimulated PBMCs in the G0/G1 phase of the cell cycle (**c**). *: *p* < 0.05; **: *p* < 0.01; ***: *p* < 0.001; ****: *p* < 0.0001.

Mesenchymal and hematopoietic markers were investigated in the freshly sorted F-SVF population and compared to the control SVF cells (Figure 5a). Data were then compared to the in-vitro-expanded ASCs to investigate the alteration of marker expression during culture time. The F-SVF cells showed no statistical difference on marker expression compared to SVF cells, proving the non-invasiveness of the methodology. There was an appreciable trend of a higher percentage of mesenchymal markers and lower CD45 expression compared to the total SVF due to a partial selection of cells from the mesenchymal compartment. The expanded ASCs indeed expressed a higher percentage of the classical mesenchymal markers CD90, CD105, and CD73.

### 3.4. Immunomodulation

The immunomodulatory properties of the cells were analyzed by two assays: measuring the proliferation of PHA-activated PBMC in contact with F-SVFs compared to control SVF cells (Figure 5b) and cell cycle distribution of PBMC in the same conditions (Figure 5c).

Both groups, SVF and F-SVF, downregulated PBMC proliferation with no differences between them, proving their immunomodulatory properties. In addition, F-SVF and SVF cells shared the same ability to stop PBMC in the G0/G1 phase (Figure 5c). This result confirmed the gentle separation process of the NEEGA-DF method and the preservation of fundamental native stem cell properties.

### 3.5. Differentiation Commitment

**Figure 6:**
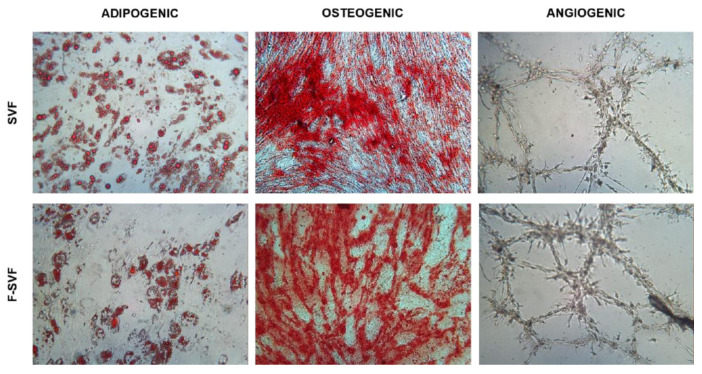
Plasticity of stromal vascular fraction (SVF) and the fraction derived stromal vascular fraction (F-SVF) cells was investigated by differentiation ability towards adipogenic, osteogenic, and angiogenic lineages. Cells from both conditions developed the characteristic lipid vacuoles (stained by Oil Red O), produced extracellular matrices (stained by Alizarin Red S), and rearranged to form tubes, as shown in the new vessel formation assay (images obtained using a 10× objective).

As a confirmation of the preservation of stem cell properties, F-SVF cells were induced to differentiate towards classical mesenchymal lineages. F-SVF cells showed the same ability to differentiate into adipogenic, osteogenic, and angiogenic lineages (Figure 6) compared to SVF cells.

## 4. Discussion

Adipose tissue is an interesting source from which to derive stem cells for regenerative medicine applications. Adipose-derived stem cells (ASCs) are part of a more heterogeneous anatomical component of the adipose tissue, the stromal vascular fraction (SVF). The SVF is derived by mechanical and enzymatical digestion of the tissue, and it is composed of a variety of cell types: mesenchymal stem cells, tissue progenitor cells, pericytes, fibroblast, adipocytes, and hematopoietic cells. Extracellular matrix proteins and red blood cells (RBCs) contaminate samples and should be discarded when the goal is the direct use of the SVF. Its direct use is increasing worldwide, especially in aesthetic and orthopedic fields, as a simpler and cheaper alternative to expanded ASCs. As clinical therapeutic applications of SVF cells continue to increase, the rapid development of cell banking is expected in future clinical scenarios, and quality control steps are mandatory to purify and control the quality of SVF cells before use. For these reasons, we investigated the use of the NEEGA-DF technique to analyze this heterogenous population due to its potential to discriminate cell population heterogeneity and, specifically, to sort and collect viable cells without the need of additional manipulation, such as antibody binding, and to discard debris and unwanted materials derived by the digestion procedure. Our results demonstrated that the NEEGA-DF cell sorting technology can be used to enrich the mesenchymal component of ASC populations from the SVF. Moreover, as an additional feature, the unwanted material contaminating the sample, such as debris from the digestion procedure, that was not eliminated by the buffer washing and centrifugation steps was eluted in the first minute of the analysis. Flow cytometric analysis of fresh SVF cells showed that a substantial proportion of cytometry events resulted from tissue debris. Their abundance depends on anatomical location, and they are usually more abundant in subcutaneous adipose tissue and, likewise, may change among patients [15]. The abundance of tissue debris probably depends on the degree of tissue fibrosis. This tissue debris overlaps the visualization of adipose tissue progenitor cells and, therefore, data can be misinterpreted unless complex staining and gating is performed [15]. Moreover, most of the RBCs elute in the void and, therefore, can be eliminated from the sample, especially because it is known that the presence of RBCs in injectable cell products can affect the therapeutic potential of stem cell treatment [16].

Current sorting technologies to isolate cells based on marker expression use pressurized sheath fluid, which passes through a narrow opening to generate droplets at a high frequency. Recently, Lopez and Hulspas proposed the term “sorter-induced cell stress” [17] because of several factors present during the cell-sorting workflow, such as sudden and repeated temperature changes, antibody activation, high pressure, shear stress, high voltage electric fields, or laser irradiation. Investigations of sorting impacts on cells are limited [10,18,19]. To overcome these issues, the NEEGA-DF method can be seen as a convenient and interesting method to sort and purify cells without the need of any additional manipulation and minimal invasiveness. The hydrodynamic forces and mobile phase rate do not affect cell viability and integrity. This simple, fast, non-invasive cell sorting methodology is able to purify homogeneous ASC subpopulations, maintaining their properties and functions unmodified. Selected cells by the NEEGA-DF method maintained their immunomodulatory ability and their plasticity to differentiate into adipogenic, osteogenic, and angiogenic lineages. Moreover, the potential of the technology lies in the enrichment of the mesenchymal cell component, as seen in the higher trend of mesenchymal marker expression and a lower CD45 expression in the F-SVF. This allowed us to obtain a cell-based product with well-defined functional properties and high differentiation potential by means of a non-invasive method with a reduced number of cell culture passages.

NEEGA-DF technology can be seen as a gentle cell sorter to enrich mesenchymal stem cells from adipose tissue and to discard debris that are often variable depending on anatomical adipose tissue origins and inter-individual variability. The “clean” SVF could then be cryopreserved for future use, since, depending on the treatment, multiple administrations of SVF will be beneficial to achieve optimal results. Instead of repeated harvesting procedures that are simple (but their repetition could increase the incidence of morbidity), to avoid patient discomfort, long-term cryopreservation could be the perfect solution to allow multiple SVF treatments. Cryopreservation adequately addressed the issue, since SVF cells can be easily frozen and stored, while maintaining their proliferative capacity and differentiation potential [20].

In conclusion, the biocompatible, highly reproducible fractionation process of the innovative technique represents a scalable opportunity to obtain a bed-side tool able to highly purify homogenous and viable ASC fractions for regenerative medicine applications.

## Data Availability

Data is contained within the article or Supplementary Materials.

## Supplementary Materials

The following supporting information can be downloaded at: https://www.mdpi.com/article/10.3390/bioengineering9040142/s1, Figure S1: Representative fractogram of expanded ASCs.
