# Supplementary figures and images for: Quality Control Platform for the Standardization of a Regenerative Medicine Product
Source: Bioengineering (Basel). 2022 Mar 28;9(4):142. doi: 10.3390/bioengineering9040142 (PMC9026409; doi:10.3390/bioengineering9040142)

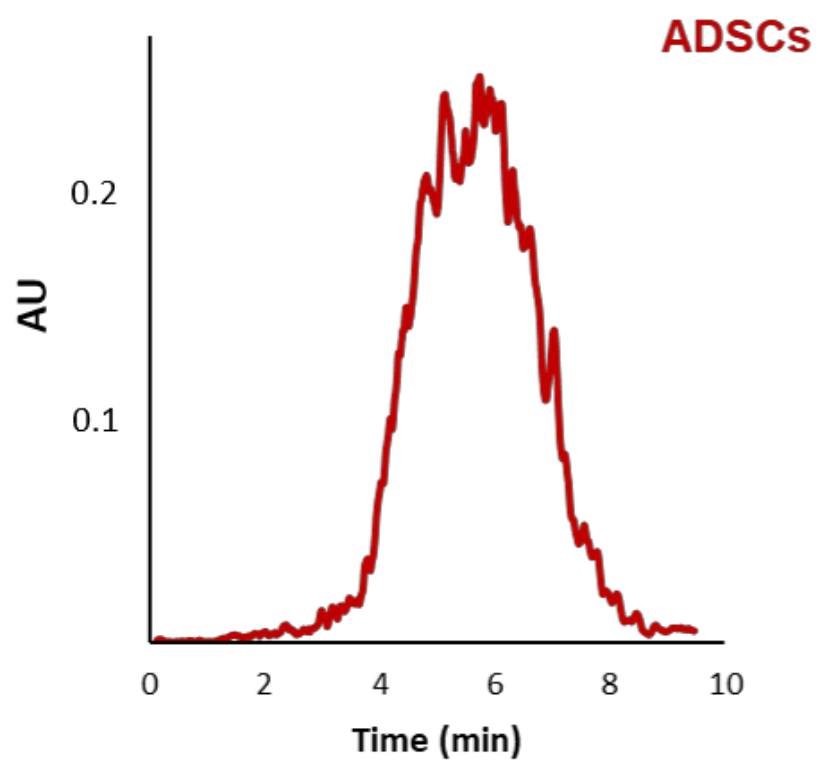

**Figure S1.** Representative fractogram of expanded ASCs.

Supplement: Supplementary file 1 [file bioengineering-09-00142-s001.zip › bioengineering-1602146-supplementary.pdf]
